# A Pilot Study of Airborne Hazards and Other Toxic Exposures in Iraq War Veterans

**DOI:** 10.3390/ijerph17093299

**Published:** 2020-05-09

**Authors:** Chelsey Poisson, Sheri Boucher, Domenique Selby, Sylvia P. Ross, Charulata Jindal, Jimmy T. Efird, Pollie Bith-Melander

**Affiliations:** 1 Emergency Medicine, SMG Norwood Hospital, Norwood (Greater Boston Area), MA 02062, USA; cpoisson_1021@email.ric.edu; 2 School of Nursing, Rhode Island College, Providence, RI 02908, USA; sboucher@ric.edu (S.B.); sross@ric.edu (S.P.R.); 3 HunterSeven Foundation, Providence, RI 02906, USA; Domenique.k.selby.ctr@mail.mil; 4 Neonatal Intensive Care Unit, Women and Infants Hospital, Providence, RI 02905, USA; 5 Joint Trauma System, Defense Center of Excellence (CoE), Fort Sam Houston, Houston, TX 02905, USA; 6 Emergency Medicine, Naval Medical Center San Diego (NMCSD), San Diego, CA 92134, USA; 7 Faculty of Science, The University of Newcastle (UoN), Newcastle 2308, Australia; charujindal@gmail.com; 8 Cooperative Studies Program Epidemiology Center, Health Services Research and Development, DVAHCS (Duke University Affiliate), Durham, NC 27705, USA; 9 Department of Social Work, California State University, Stanislaus, Stanislaus, CA 95382, USA; polliebith@gmail.com

**Keywords:** burn pits, Iraq War, physical health, toxic exposures, Veterans

## Abstract

During their deployment to Iraq in support of *Operation Iraqi Freedom (OIF)*, many Veterans were exposed to a wide array of toxic substances and psychologic stressors, most notably airborne/environmental pollutants from open burn pits. Service members do not deploy whilst unhealthy, but often they return with a multitude of acute and chronic symptoms, some of which only begin to manifest years after their deployment. Our findings, while preliminary in nature, suggest that Iraq War Veterans who participated in our survey reported a decrease in overall physical fitness and increased respiratory clinical symptoms compared with pre-deployment periods. The objective of this report is to provide information that will benefit how combat Veterans are cared for post-deployment. Strategies for a wider and more comprehensive assessment and medical screening process post-deployment are recommended.

## 1. Introduction

Over two million United States service members have been deployed to Iraq since 2003 in the continuing support of “*Operation Iraqi Freedom (OIF)*” [1]. Increased rates of respiratory illnesses, debilitating physical ailments, and rare forms of diseases have been observed in troops after returning from tour duty in Iraq [2]. In many cases their symptoms resemble those seen in much older individuals, rather than the previously robust and physically fit recruits at the time of deployment. This is consistent with earlier reports of ‘military accelerated aging’ associated with Veterans exposed to toxicants during the Gulf War I conflict [3,4,5].

Airborne toxins represent the main class of exposures reported by those who served in the Iraq theater of war and counterinsurgency effort [6,7]. This includes contaminated particulate matter from frequent dust storms, combat-related smoke from ground ordnance and air strikes, as well as near-field (on-base) contact with oil-well fires, diesel fumes, and open-air burn pits [2]. A “*burn pit*” describes a constructed hole in the ground used to dispose of environmental garbage and military waste [8,9]. The pit is ignited with various accelerants, and other substances to aid the spread of flames. Often the fire may smolder around-the-clock for weeks to months, posing a lasting health concern to exposed servicemen and women. Prevailing winds further spread airborne contaminates to outlying areas miles from the source. 

American forces operated more than 250 burn pits on Joint-Base Balad, Iraq to accommodate the accumulating waste of warfighters and support personnel stationed in the region [6]. An estimated 140 tons of trash was burned daily, with the typical soldier contributing 10 pounds per day (Reiss, 2012). In an official assessment conducted by the Air Force in 2006, the burn pit at Joint Base Balad was described by the author as “the worst environmental site I have personally visited” [10]. The report documented the burning of high quantities of various toxic materials (e.g., plastics, tires, paints, batteries, Styrofoam™, medical waste, electronic equipment, pesticides, and human trash) using the jet fuel propellant (JP-8) as an accelerant [10]. Compared with standard petroleum-based fuels, JP-8 adheres more effectively to contact surfaces for a longer period, extending the health risk of exposure [11,12]. A common complaint of this pungent and ‘oily to the touch’ compound is a lingering smell and taste of JP-8 for hours after exposure (author experience). Recognizing the potential chronic health hazards associated with burn pit smoke, the report recommended that military personnel stationed at Joint-Base Balad have a permanent memorandum placed in their medical record of the exposure risk [10].

The aim of this preliminary research is to bridge gaps in knowledge for Veterans and healthcare providers and facilitate strategies for a more complete and comprehensive medical screening processes for previously deployed Iraq Veterans. The information presented on clinical symptoms and overall physical fitness test scores before and after deployment will be helpful for planning future studies of this vulnerable Veteran population and establishing the fidelity of participant recruitment and data collection. Our findings, conclusions, and recommendations also are relevant to other theaters (downrange environments) and not just for American forces. While the focus of this article is Iraq, it is an example of a larger and more widespread problem with global relevance.

## 2. Materials and Methods 

### 2.1. Participants 

This study used a point-of-reference survey of military combat Veterans who served in OIF between March 2003 and December 2011. Those who served outside of Iraq (e.g., Afghanistan, Kuwait) or exclusively during a different conflict period (i.e., 1990–1991) were excluded. However, a small number (<10%) of Veterans who previously served in the Gulf region participated in the survey. To address participant’s eligibility status, the questionnaire opened with “*Have you served with the United States Military in Iraq between March 2003 to December 2011?”.* This question was intentionally placed before allowing access to consent or additional questions. 

### 2.2. Materials and Procedure

Participants were informally recruited through Veteran websites, newsletters, chatrooms, Twitter, and Instagram from 1 July to 21 October 2018. In some cases, participants self-referred by contacting Veterans who were active (or listed) on various on-line sources (e.g., https://linktr.ee/huntersevenfoundation; https://www.publichealth.va.gov/exposures/gulfwar/); https://www.instagram.com/huntersevenfoundation/?hl=en; https://www.wehonorveterans.org/).

A 66-item internet-based questionnaire was used to collect data, with participants completing the survey at their own discretion. No compensation was offered. The study was approved by the collegiate Institutional Review Board, and all ethical standards were maintained. The average survey completion time was 28 min. The full study questionnaire may be requested from the HunterSeven Foundation (info@HunterSeven.org) subject to use and distribution restrictions.

### 2.3. Measures and Covariates 

Participants were asked basic demographic questions (e.g., age and sex), as well as their health status, and Iraq War designation. Exposure and epidemiologic variables were adapted from the Department of Veterans Affairs *Airborne Hazards and Open Burn Pit Registry* and included for example the type of burning source (burn pit, small barrel fire, incinerator) [13]. Items in this registry have been previously reviewed for psychometric properties such as consistency and accuracy. While information pertaining to the specific materials, volume, intensity, and timing of the burn source was not collected, questions were included about deployment duration (time in country), distance of sleeping/housing and workstations from burn pits, frequency of exposure to smoke/fire, and exercise (miles run). In the case of certain questions such as physical activity level, pre- and post-assessment responses were solicited.

### 2.4. Statistical Analysis

Given the pilot nature of the study, results were largely presented descriptively as percentages/averages, without confidence intervals or standard errors of measure. 

## 3. Results

### 3.1. Demographics

The average age of participants at the time of survey was 37 years (N = 109), with a standard deviation (SD) of 7.5 (Table 1). Most participants (82%) identified as male. Thirty percent were “*still currently serving in military*”, with the average time in service being 6 to 8 years (21%) and 20+ years (18%). The average paygrade/military rank was E-4 through E-6 (70%). 

An increase in “situational use” was observed for tobacco and nicotine products, with 42% reporting use while deployed, compared with 22% post-deployment. Forty-one percent of respondents reported having “*new allergy-like symptoms*”, and 35% underwent a diagnostic polysomnogram in response to complaints of disrupted sleep, excessive daytime sleepiness/napping, lack of energy, anxiety, and depression post-deployment. The percentage of respondents who requested a sleep study was noticeably higher when compared with 6% of the American population with a diagnosis of “sleep apnea” [14]. Among post-deployed Veterans, a polysomnogram is helpful to differentially diagnose sleep apnea from other sources of interrupted sleep such as chronic pain from war-related injuries, medications to treat premature comorbidities, and psychiatric conditions related to their military service (e.g., post-traumatic stress disorder, severe nightmares, excessive alcohol or caffeine use). 

Furthermore, 34% of respondents reported undergoing a “pulmonary function test” which measures the adequacy of lung function. Comparatively in the civilian setting, pulmonary function tests are mainly reserved for adults over age 55, asthma patients, and those with chronic lung diseases” [15]. See “Appendix A (Table A1)” for additional “demographics” [16,17].

### 3.2. Symptoms and Deployment(s)

All deploying service members are required to complete Department of Defense Form 2795, a Pre-Deployment Health Assessment. This provides an opportunity for a medical provider to address and evaluate medical readiness. Prior to military service in Iraq, 80% of respondents reported having “*no symptoms*”. Those who reported symptoms included*: acid reflux, headaches, joint/muscle pain, dizziness, insomnia* and *memory loss*. Additionally, military guidelines and regulations require a satisfactory physical fitness status in order to deploy. All the participants in the study stated they had a “*passing*” physical fitness test score prior to deployment to Iraq.

One of the survey questions asked participants to state how many “*combat deployments*” they’ve encountered in order to determine any variations in medical and fitness status in relation to number of exposures (repeated vs. single). Forty-four percent reported “one”, 24% reported “two”, 20% reported “three” and 11% reported “four-to-six” combat deployments. Additionally, the year(s) of those deployments were clarified to determine if a certain period was more causative of symptomology than others and if increased and/or decreased overall combat operations had an impact on health status and physical fitness. Many respondents in our survey deployed to Iraq in 2004, 2005, and 2007, with numbers of deployed troops decreasing in 2009 to 2011 (4–9%), reflecting policy changes. 

Ninety-seven percent of respondents stated, “*burn pits*” were the primary method of waste disposal, 58% used “*small barrel fires*” and 9% stated an “*incinerator*”. Thirty-five percent reported an approximate distance to burn pits from housing within ¼ of a mile, 26% reported within 100 yards with similar trends in distances in working areas to burn pits/barrel fires. 

Joint-Base Balad had been one of the largest military instillations in Iraq, and data shows that in 2007, this base was burning up to 240-tons of waste per day (Reiss, 2012). The question was deemed imperative to determine the percent of our respondents who have been to “*Joint-Base Balad*” (also known as “*Balad Air Base*”) while in Iraq. Eighty eight percent of our respondents stated they had passed through this military hub at one point during their deployment.

**Figure A1:**
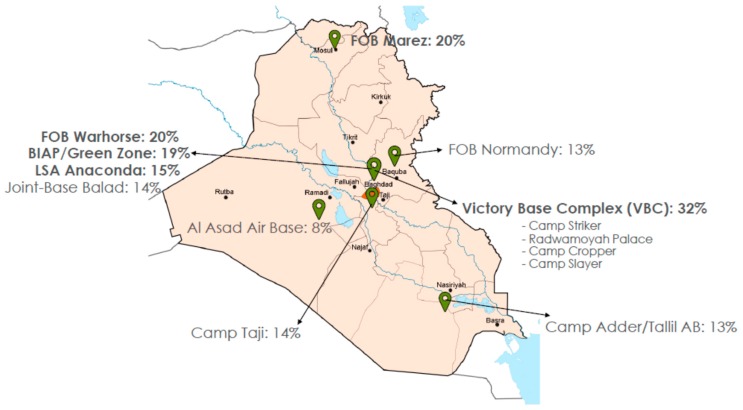
All Deployment Location(s). FOB: Forward Operating Base, BIAP: Baghdad Intl. Airport, LSA: Logistics Supply Area, AB: Air Base.

To determine trends in adverse physical conditions, respondents were asked to disclose which military instillations they had spent most of their time deployed at. Locations of bases accounted for altitude, shamal winds (predominately originating from northwest and having the greatest intensity during the summer and daytime), and other environmental considerations in relation to toxic exposures. These instillations include: Victory Base Complex, Forward Operating Base (FOB) Warhorse, FOB Marez, Baghdad International Airport (BIAP) also known as “*the Green Zone*” and Logistics Supply Area (LSA) Anaconda (Appendix B, Figure A1 and Table A2). 

**Figure 1:**
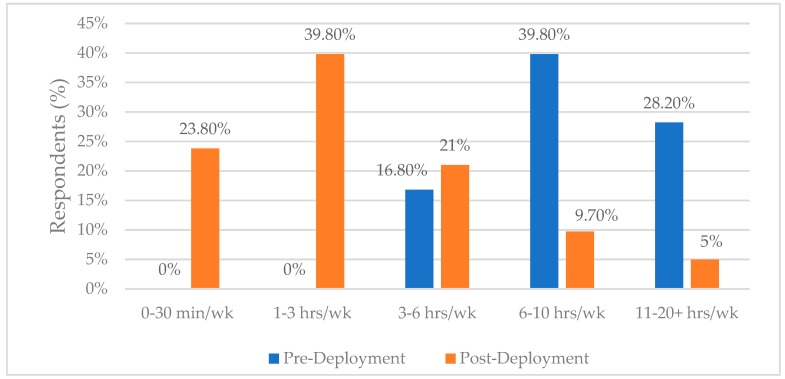
Physical Training Conducted.

Figure 1 displays the percentage of respondents who conducted hours per week of physical fitness training in both pre-deployment (blue) and post-deployment (orange). The data displayed shows participants conducted longer durations of physical exercise weekly pre-deployment bringing forth the question, did duration of physical training decrease owing to physiological changes and physical conditions?

To understand toxicity in relation to exposures, respondents were asked which items they witnessed being burned or personally burned themselves. Highest response rates included plastics, papers, cardboard items, Styrofoam™, military clothing, treated wood/pallets, oils and fuels. In addition, respondents noted high rates of potential exposures to other sources as well, such as diesel/JP-8 fuels, smoke/soot from burning wastes, insect bites, Iraqi vehicle emissions, generator/tent smoke, pesticides, and Iraqi ordinance. 

**Figure 2:**
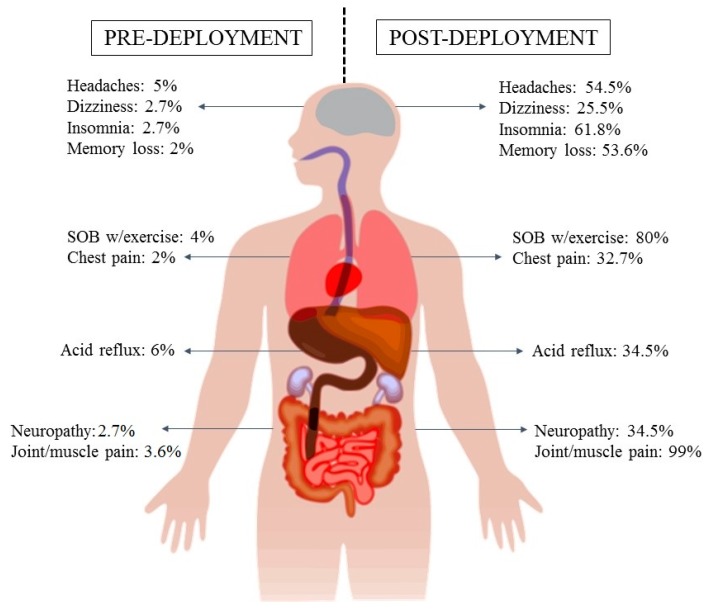
A Pictorial Depiction of Pre- and Post-Deployment Health Status of Iraq War Veterans (SOB = shortness of breath).

Figure 2 shows the increased percentages in reported symptoms in the pre- and post-deployment stages. Respondents were asked to report only “chronic” symptoms with the stated period defining “chronic” being 1-month in duration. The highest rates of change were observed for joint muscle pain, shortness of breath with exercise, insomnia, headaches, and memory loss. Fatigue (not depicted in the table) also was higher following deployment. Other symptoms of importance include those which were present while deployed but decreased shortly after leaving Iraq and the combat environment such as loss of smell, burning eyes and throat, coughing spells, irritated throat, reoccurring nosebleeds, nausea, vomiting, and discolored phlegm. 

**Figure 3:**
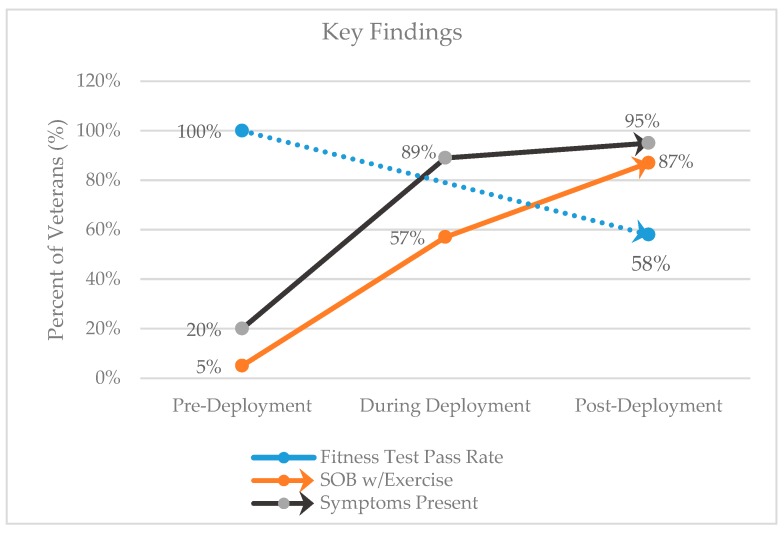
Respiratory related symptoms.

Participants in general reported an increase in respiratory related symptoms along with a decrease in physical fitness status. The most imperative data (Figure 3) are displayed in three essential forms, fitness test pass rates, shortness of breath with exercise and Veteran reports of respiratory related symptoms (20% to 89% during deployment and up to 95% post-deployment).

## 4. Discussion

### 4.1. Overview

To our knowledge, this is the first health outcomes survey of OIF Veterans conducted by a peer-support organization. Preliminary analyses suggest that Veterans who served in this conflict and were exposed to various airborne toxins consequently manifested increased respiratory related symptoms and decreased physical fitness status. 

Many of the increased symptoms associated with deployment are considered in the medical community as “*acute*”, meaning an external source acts as an irritant creating an allergen-cascade reaction. This includes forming B-cells and Immunoglobulin-E (IgE) antibodies which react to the foreign antigen in the form of symptoms such as loss of smell, burning eyes and irritated throat, coughing, and discolored phlegm [18]. Upon leaving Iraq and removing the irritant(s) causing the reaction, those specific symptoms showed a decrease. Nonetheless, concerns remain regarding the long-term health outcomes of such uncontrolled airborne exposures and our results emphasize the need for post-deployment follow-up and continued monitoring of this high-risk group. They also point to policy changes that are necessary to mitigate risk in future deployments. For example, a more suitable method of waste disposal in war zones, such as incinerators, would in theory decrease experienced symptoms and health risks.

### 4.2. Existing Literature on Long-Term and Persistent Effects of Exposure

Like military personnel deployed to *Operations Desert Shield and Desert Storm*, Veterans of OIF were exposed to a multitude of hazardous agents and psychologic stressors and have since reported symptoms in multiple body systems [19]. The effect of these exposures has been both acute and long-lasting. In some cases, the presentation and reporting of symptoms is delayed for many months to years, only first coming to the attention of medical providers when coupled with other life-course events, comorbidities and aging [4]. 

Several underlying mechanisms have been postulated for the long-term deleterious health effects of Veterans who were deployed to the Gulf region. Biologic evidence comes from a study of mitochondrial DNA damage of Veterans exposed approximately 25 years in the past and now exhibiting >3 domains of moderate-severe illness (i.e., fatigue, pain, neurologic, cognitive, mood, skin, gastrointestinal, and respiratory) [20]. Compared with an unexposed military population, exposed Veterans manifested 20% higher levels of mtDNA damage (*p* = 0.015) along with non-significant increases in nuclear DNA lesion frequency. Greater mtDNA damage is consistent with mitochondrial dysfunction (secondary to environmental and chemical toxicants) and offers supporting pathophysiologic evidence for the persistence of illness over time. Independent of smoking history, subsequent effects also are believed to impact the efficiency of electron transport chain complexes to clear reactive oxygen species from the body.

A range of chemicals and toxins are thought to impair mitochondrial and basic cellular functions for days to years after exposure, impeding the body’s ability to detoxify these agents [21,22]. Coenzyme Q10 (CoQ10) is a potent antioxidant that aids mitochondrial energy production. In a randomized, double-blind trial of Veterans with symptoms thought to be the result of their service-related exposures during deployment to the Gulf region (e.g., muscle weakness, fatigue with exertion, cognitive impairment, gastrointestinal ailments, and skin problems), those who received CoQ10 versus placebo had improved physical function (summary performance scores) [23]. Furthermore, the level of benefit was correlated with increasing concentrations of CoQ10 in the bloodstream. 

DNA damage and mitochondrial repair, especially in the context of inductive-adaptive protection, remain a complex topic. The persistent clinical symptoms associated with deployment may reflect an inability to return to an uninduced hormetic state after the elimination of putative exposures, resulting in a “long-term metabolic shift” [24]. This process likely is a function of the extent and duration of exposure in combination with endogenous genetic regulation, with some Veterans effected more severely than others.

In another study postulating long-term effects of deployment to the Gulf region, Veterans had increased Bacteroidetes, but decreased Firmicutes in their microbiota, compared with a non-effected referent group [25]. In general, as noted by the authors, imbalances in the human microbiome have been associated with neurologic disorders, inflammatory bowel disease, metabolic disorder, liver disease, and cancer. Furthermore, experimental results conducted by the same team in a murine model reinforced human findings. Animals had altered enteric viral populations in the gut, indicative of inflammatory phenotype and neuronal immunotoxicity [26]. While these initial results are provocative, more definitive studies with a larger cohort and adjustment for antibiotic use history are needed to validate long-term alterations in gut-microbiome among this group of Veterans, especially given their higher documented rate of infection [25]. 

The constellation of health issues experienced by Iraq War Veterans appears similar in many respects to the earlier cohort of Gulf War I Veterans, consistent with chronic multisymptom illness (CMI) (personal communication with Dr. Steven C. Hunt, Director, Deployment Health Clinic, VA Puget Sound Health Care Systems). In a prospective longitudinal study, approximately half of *Operations Iraqi Freedom/Enduring Freedom* (OIF/OEF) Veterans met the criteria for mild to moderate CMI. Additionally, 11% fulfilled the case definition for severe CMI, while 90% of those with chronic musculoskeletal pain met the criteria for CMI [27]. Nonetheless, the symptoms most common to OIF/OEF Veterans were not necessarily those most often observed among the Gulf War I cohort, suggesting overlapping but not equivalent conditions. For example, while nearly all Veterans who served in Iraq and Afghanistan experienced at least one environmental hazard, the most common exposure was to air pollution and pesticides, precipitated by sandstorms and burn pits [28]. In the case of airborne hazards, many of the deployed military personnel to OIF/OEF were exposed to levels that far exceeded safe exposure guidelines [29]. CMI is a highly nonspecific diagnosis and to some extent discordant characterization for the illnesses reported by Veterans who were deployed to the Gulf region. Nonetheless, this term remains a basic starting point when assessing the varying and multiple exposures experience by Veterans deploy to this region. 

The Millennium Cohort reported increased odds of CMI (odds ratio = 1.70) among those who were deployed to combat duty in Iraq and Afghanistan compared with pre-deployment assessment [30]. While the prevalence of symptom reporting increased across all Millennium Cohort Study groups during the second time interval between surveys (2004–2007), suggesting a persistence of symptoms, this trend could alternatively be attributable to multiple deployments by the end of the second time interval.

The toxic exposures experienced by Veterans of the Wars in Iraq and Afghanistan appear to have lasting effects, and for nearly 40% of Veterans, it is a syndrome that is similar in presentation to CMI [28]. Notably, this finding appears to hold even after accounting for key confounding variables known to be associated with post-deployment somatic symptoms. Given the cascade of symptoms reported after deployment to the Gulf region, independent of the year period, researchers have hypothesized that CMI is not attributable to a single stressor but rather is the consequence of exposure to several different types of exposures over a relatively brief time window [31]. In retrospect, the illnesses associated with the above-mentioned military conflicts likely mirror similar chronic conditions such as fibromyalgia and chronic fatigue syndrome, which also are believed to have abstruse (and possibly multiple) etiologies [31]. While definitive supporting evidence is lacking, idiopathic environmental intolerance may possibly explain the equivocal nature of such conditions. Under this unifying framework, both acute and long-lasting symptoms of nonspecific but multiple organ systems, are hypothesized to be the result of subtoxic exposure to an amalgamation of chemicals and/stressors [32,33], resulting in chemical sensitivities [34]. The accumulated body burden of the latter may underlie why certain Veterans do not have uniquely high exposure levels of a single toxin or single event-related exposure, but rather they represent a complex mixture of xenobiotics and genetic predisposition [35]. Interestingly, in a systematic review, Veterans of the Gulf War were approximately three and a half times more likely to report multiple chemical sensitivity or CMI as defined by the Centers for Disease Control (CDC) [36]. 

Another potential mechanism underlying the persistent CMI symptoms experienced by OIF Veterans is a primed inflammatory response vis-à-vis insecticide exposure [37]. Various insecticides, including agents obtained on the local market (such pet tick-and-flea collars), were used in OIF to combat sand flies [38]. In many cases, the compounds in the form of liquid were regularly applied to the skin or included as waste in burn pits and have been hypothesized as the basis for ‘sickness behavior-like symptoms’ reported in the literature [37]. 

### 4.3. Unique Aspects of the Iraq War 

The Iraq War presented with its own unique history and characteristics. For example, an important consideration when assessing the epidemiologic risks attributed to the Iraq War is the “shock and awe” theory [6]. This framework posits that thousands of pounds in hazardous agents, such as burn pit residual, military waste, and chemical weapons, accumulated or leaked into the sand over the course of the current and previous deployments to the region, and thus posed a cumulative inhalation/contact hazard. Iraq also differed from other theaters of war in terms of prevailing winds, climate, topography, and altitude, factors which may have uniquely impacted risk. 

### 4.4. Unknown Threats to Health and Perceived Barriers to Care

Combat casualty rates were at an all-time high in 2004, 2005, and 2007 in Iraq [39]. Most respondents in our survey reported deploying in 2004, 2005, and 2007, respectively. During deployment to a combat zone, service members do not focus solely on environmental exposures and airborne toxins, but more so on the combat environment itself and resulting injuries. Secondly, understanding that military guidelines and regulations are exact and authoritative, it is perplexing that such activities would occur that would place the service member at risk. Awareness briefings and literature; appropriate pre-deployment training related to the matter; attention to risks and enforcement of risk reduction (through protective measures, e.g., appropriate clothing and masks/filters/respiratory equipment) were minimal. Personal protective equipment (PPE) as evidenced by the questionnaire was either not issued or not used by service members. Utilizing respirator masks while working in the immediate area(s) of burn pits would decrease service members’ potential exposure to environmental toxins. However, and understandably, when deployed to a combat zone, consideration of environmental exposures and airborne toxins are generally sidelined. Service members have other more critical issues to focus on such as life-threatening injury and survival. 

Perceived barriers to care post-deployment includes risk of loss of job (military) and risk of medically related military discharge (IAW AR 40-501: Standards of Medical Fitness, AR 600-60: Physical Performance Evaluation System, AR 635-40: Physical Evaluation for Retention, Retirement and Separation). Most respondents fell into the 25–34-year-old range and 30% are still currently serving in the military. Often Veterans assume that if they seek treatment for chronic symptoms they may become “flagged” and removed from their military job or discharged from service which would have directly impacted many respondents. This includes disincentive or negative incentive aspects (sociologic/psychologic factors) for resisting treatment. In addition, our average age group did not fall into the “common” health concern cohort, i.e., those chronic conditions more commonly seen in the older population versus a previously healthy, physically fit service member. One example is the earlier onset of acute myeloid leukemia in the Iraq War Veteran population resulting in death before age 40, compared with the national average age for this specific cancer of 70 years [40]. Because of this, some service members may avoid seeking medical treatment. Additional barriers of care include those who utilize civilian healthcare facilities versus the Department of Veterans Affairs Medical Centers. When an Iraq War Veteran is seen at a civilian hospital for respiratory-like symptoms, the civilian providers, unfamiliar with the toxicity of burn pit exposure, may attribute the symptoms to tobacco use, lack of exercise, allergies, or bacterial and viral infections. However, if the same Iraq War Veteran is seen at the Department of Veterans Affairs Medical Center, the respiratory-like symptoms may be viewed differently; the possibility of toxic exposures during deployments is a known entity. 

### 4.5. Nursing Implications

Professional nurses play an important role in the interprofessional health care team particularly with patient assessment, education and navigation. As discussed above, when a person enters the healthcare system, assessment frequently starts with a general knowledge of typical age-related concerns and patterns. The healthcare provider would see a younger, fit adult and may assume the underlying cause of respiratory symptoms is an acute infection or an irritation from lifestyle factors such as smoking or seasonal allergies. However, triage and/or assigned nurses who include the simple question “*Are you a Veteran*”, the initial algorithm related to respiratory symptoms diagnosis and patient care would have a different interpretation. The healthcare team could consider the potential cause for respiratory symptoms as one that is related to chronic respiratory difficulties associated with toxic environmental exposure to burn pits while deployed, especially in Iraq. Professional development for nurses and other healthcare providers on the toxic effects of burn pit exposure in Veterans who have served in Iraq is the responsibility of healthcare administrators, in both civilian and military hospitals. 

Military nurses perform a significant role in educating service members about the potential risks associated with deployment to another country or region of the world. Educating soldiers and other military personnel about burn pits and hazardous exposures could start with emphasis on the use of personal protective equipment (PPE), and the benefit of decreased exposure risk. Pre-deployment briefings before deployment to zones that utilize burn pits would increase awareness of the potential exposures as well as post-discharge/retirement and Veteran screenings. Theoretical support for this approach is supported by the Health Belief Model (HBM), as described below.

In general, they have more frequent and intense engagement, both in theater and post-deployment, in serving the healthcare needs of Veterans. Nurses with military field experience especially have the greatest impact (i.e., most important in preventive aspects as well as identification and treatment) and serve a critical role in helping to resolve problems, finding practical solutions, and improving the operational effectiveness of post-deployment healthcare. They also are best positioned to aid the mission of the U.S. Army Training and Doctrine Command (TRADOC) to ensure the safety and battlefield fitness of our warfighters and their healthcare needs afterwards. 

### 4.6. The Health Belief Model

Key components of this conceptual framework are grounded in social psychology. The underlying premise is that individuals (in this case service members) are more likely to engage in health behaviors when they more fully understand the susceptibility to the risk, the seriousness of the risk and potential consequences, which specific beneficial actions they can take to prevent risk and exposure, and when the perceived barriers or risks outweigh the avoidance of action [41]. If service members were alerted to the existence of the burn pits, and how to decrease potential harm from exposure (wearing protective gear as much as possible), perhaps the long-term sequelae would be less [42]. To assist in alerting, acknowledging and providing education, registered nurses could screen for Veteran status, identify modifiable variables, and determine perceived susceptibility, seriousness and threat. By determining the Veterans’ perceived barriers and benefits, self-efficacy, and potential cues-to-action, the nurse can assist in educating in health promotion, behavioral changes in addition to screening for Veteran-specific illnesses. 

### 4.7. Limitations

Most respondents served in one military branch (Army) and were enlisted, “non-commissioned officers”. Accordingly, our results may not generalize to all Iraq War Veterans or to other military populations. The study relied on “*self-reported*” data, with a potential increased risk of biases, attribution, exaggeration, and selective memories. We also are unable to rule out that sicker Veterans may have responded to our survey. While exposures to certain toxic substances, even at low levels or for brief periods, may increase disease risk, information pertaining to the level, intensity, and mode of contact with such putative agents was not collected in this study. The unique genetic vulnerability of a Veteran to a particular risk factor was unknown, as was the exact etiology and pathobiology of symptoms many years after the putative exposure(s). Inconsistencies regarding CMI symptom reporting for burn pit exposures across studies may have limited the generalizability of findings [38,43]. Some ambiguity exists in how physical fitness was assessed in our survey. Further versions will benefit by being able to differentiate if observed decreases in physical fitness were attributable to time spent or shortness of breath during exercise. 

As is the case for a single-point-in-time survey, Veterans who died were not included in the analysis suggesting that the rates for some outcomes may be underreported. However, our sample was not restricted to participants who only used Veteran Administration facilities for their health care. Previous assessments of this specific cohort were not available for comparison. 

The key authors of this manuscript are Veterans who were deployed to Iraq in support of OIF. In establishing the non-profit HunterSeven Foundation, they hoped to provide an independent resource for fellow Veterans to express their experiences. The initial survey information collected by the Foundation was not intended to be a research project but rather a means to acknowledge and explore potential exposures associated with their deployment and a vehicle for encouraging Veteran engagement and communication. While it lacks a certain degree of epidemiologic rigor, the participants were more candid and willing to provide insights that often are missing in more formal surveys conducted by the Veterans Administration (VA) or the Department of Defense (DoD). In the future, it is hoped that the HunterSeven Foundation will serve as a recruitment resource for difficult to reach Veterans of this conflict. In every sense, the data collected thus far by the HunterSeven Foundation is preliminary in nature. The sample size is small, and resources have not been available for follow-up data cleaning or to recontact Veterans regarding incomplete or inconsistent survey responses. Because of the before and after deployment context for several survey questions, there are many cases of missing values. If the sample size was larger it would be feasible to impute or model the missing data. Accordingly, traditional statistical tests for comparing correlated (before-after) data and obtaining p-values were not performed but rather only simple percentages were reported. Nonetheless, it is hoped that the information presented will be helpful in planning the future recruitment of this population. For interested readers, the data reported as percentages (p) follow a binomial distribution and the standard deviations may be estimated as the square root of p(1 − p)/N. For crude comparisons of before and after groups, one may use these standard deviations, keeping in mind that the underlying data is correlated and that such comparisons will not be statistically rigorous (and thus are not provided in the manuscript).

### 4.8. Future Research

Areas for future research include the amount of time service members were exposed to harm, the contents burned on specific bases, and whether time since their deployment impacted the health status of Iraq War Veterans. Research also is needed to determine if wearing protective gear (and the recycled use of such equipment) when directly exposed to burn pits decreased (or increased) acute and long-term adverse health effects. Lastly, gene–environment interactions likely contributed to the manifestation of symptoms among selected OIF Veterans and merits further exploration. 

## 5. Conclusions

Our preliminary findings suggest that Iraq War Veterans who participated in this survey had a decrease in overall physical fitness and increased respiratory clinical symptoms, compared with pre-deployment to post-deployment periods. The objective of this report is to provide information that will benefit how combat Veterans are cared for post-deployment and their health recovery. Strategies for a wider and more comprehensive assessment and medical screening process post-deployment are recommended. 

## Figures and Tables

**Table 1:** Demographic characteristics of participants at the time survey.

Age (mean)	37 years
Gender (Male)	82%
Still Serving	30%
New-onset Allergies	41%
Polysomnogram	35%
Pulmonary Function Test	34%

## Appendix A

**Table A1:** Demographic Data Table.

Demographic	Response	Respondents (%)	National Data (%) *
Age	25–34 years	42	49
	35–44 years	41	23
Gender	Male	84	88
	Female	16	12
Blood Type	A+	37	34
	O+	36	38
Allergies	Yes	41	40
	No	59	60
Branch of Service	Army	84	54
	Navy	5	17
	Marine Corps	7	14
	Air Force	6	15
Currently Serving	Yes	29	n/a
	No	71	
Avg. time in Service	6–8 years	21	n/a
	14–16 years	17	
	20+ years	18	
Avg. Rank	E-5	27	Enlisted: 85
	E-6	24	
	E-4	19	
Avg. MOS **	Infantry	21	n/a
	Mechanic	15	
	Mil. Police	9	
	Field Artillery	8	
	Transportation	8	
MOB Station ***	Rose Barracks, GE	13	n/a
	Camp Shelby, MS	9	
	Fort Dix, NJ	8	
	Ft. Lewis, WA	6	
	Ft. Bragg, NC	6	
Immunization(s)	Anthrax	90	n/a
	Small Pox	89	
	H1N1 Influenza	66	
	Anti-Malaria	56	
Number of Deployments	1-deployment	44	57
	2-deployment	24	27
	3-deployment	21	10
Smoking/Nicotine Use	Current Smoker	22	n/a
	Smoked during Deployment	42	
	Non-Smoker/Never	36	
Exercise Stress-Test Post-Deployment	No	55	n/a
	Yes	34	
	Unknown	11	
Sleep Apnea Study Test	No	64	n/a
	Yes	34	
	Unknown	0	

*Note*: * - “National Average” accounting for the Iraq War Veteran Population as a whole, nationally. ** - “MOS” abbv. for military occupational specialty, the job duties assigned to servicemember. *** - “MOB” Station is the mobilization station/last duty station prior to deployment.

## Appendix B

**Table A2:** Base Name and Location.

Base Name	Location	Respondents (%)
Victory Base Complex	Baghdad, Iraq	32
FOB Warhorse	Baqubah, Iraq	20
FOB Marez	Mosul, Iraq	20
BIAP/Green Zone	Baghdad, Iraq	16
LSA Anaconda	Baghdad, Iraq	15
Joint-Base Balad	Balad, Iraq	14
Camp Taji	Taji, Iraq	14
FOB Normandy	Baqubah, Iraq	13
Camp Adder/Tallil AB	Tallil, Iraq	13
Al Asad AB	Al Asad, Iraq	8
Camp Fallujah	Fallujah, Iraq	8
Camp Falcon	Baghdad, Iraq	7
Camp Speicher	Tikrit, Iraq	6
FOB Q-West	Qayyarah, Iraq	3
